# Impact of education and income inequalities on life expectancy: insights from the new EU members

**DOI:** 10.3389/fpubh.2024.1397585

**Published:** 2024-08-21

**Authors:** Gamze Sart, Yilmaz Bayar, Marina Danilina

**Affiliations:** ^1^Department of Educational Sciences, Hasan Ali Yucel Faculty of Education, İstanbul University-Cerrahpaşa, İstanbul, Türkiye; ^2^Department of Public Finance, Faculty of Economics and Administrative Sciences, Bandirma Onyedi Eylul University, Bandirma-Balikesir, Türkiye; ^3^Department of Economics, Plekhanov Russian University of Economics (PRUE), Moscow, Russia; ^4^Department of Economics, Financial University under the Government of the Russian Federation, Moscow, Russia

**Keywords:** educational inequality, income inequality, ICT indicators, CO_2_ emissions, real GDP *per capita*, life expectancy, panel regression analysis

## Abstract

Life expectancy is one of the primary population health indicators and in turn increases in life expectancy indicate improvements in population health and human welfare. Therefore, one of the ultimate goals of the countries is to increase the life expectancy. This article studies the effect of education and income inequalities, ICT indicators, CO_2_ emissions, and real GDP *per capita* on life expectancy in the new EU members for the period of 2010–2022 by employing fixed effects regression. The coefficients of panel regression uncover that education and income inequalities and CO_2_ emissions negatively impact life expectancy, but ICT indicators of internet usage and mobile cellular subscriptions and real GDP *per capita* positively affects the life expectancy. The findings of the panel regression analysis indicate that public policies to decrease the inequalities in education and income will make a contribution to life expectancy.

## 1 Introduction

Human capital is one of the fundamental elements of economic growth and development process. The physically and mentally healthier employees are expected to be more productive and show higher performance (1), and employees with lower health status generally take more time off work owing to the illnesses (2). Therefore, it is vital for the countries to experience improvements in population health, defined as the health outcomes of the individuals in a society (3), given the economic roles. Furthermore, good health and well-being is one of the 17 sustainable development goals (SDGs) and good health is crucial to achieve the remaining SDGs (4).

However, significant heterogeneity exists among population health levels proxied by life expectancy and inequalities in life expectancy of the countries in the world. For example, there was a 31.823-year gap in life expectancy at birth between Japan and Chad in 2022 and inequality in life expectancy in Slovenia and Nigeria was 2.1491 and 39.7452 in 2022, respectively, (5). But the globally convergence of life expectancy and inequalities in life expectancy among the countries appears to be quite slow. Health inequalities can lead many socio-economic costs such as higher health care costs, losses to productivity, economic growth and development, and welfare (6). Therefore, economic and social inequalities within and between countries are increasing further as a result of health inequalities. In this context, exploration of the factors underlying remarkable differences in life expectancy of the countries is important to arrange the optimal policies for the convergence of life expectancies of the countries and achievement of the SDGs.

The empirical studies have suggested many institutional, socio-economic, and environmental factors to promote the population health. Education, income level, employment, infrastructure, environmental quality, institutional quality, nourishment, social expenditures, clean water, sanitation, health sector development (health expenditures, physician density, hospital bed density), medical technology development, and financial development can be listed as the most important factors (7–9). This article analyzes the impact of inequalities in education and income along with information and communication technologies (ICT) indicators, CO_2_ emissions, and real GDP *per capita* on life expectancy regarding the gap in the empirical literature explained in Section 2.

In this regard, education can impact life expectancy through multiple direct and indirect channels. First, people with higher education level usually have more health awareness about three ingredients of public health including prevention, protection, and promotion and more information about healthy lifestyle in terms of nutrition, mental and physical activities (10). Secondly, persons with higher education generally have better-paid jobs and in turn higher life quality and medical care (11). Thirdly, education can enhance life expectancy through improvements in production of health services (12). Last, education can affect life expectancy through economic growth and development because education is a key driver of human capital, a significant determinant of economic growth and development (13–15). In conclusion, it is expected that education positively affects life expectancy through the aforementioned multiple channels, but inequalities in education can negatively impact the life expectancy by distorting the interaction between life expectancy and education.

Income level can also affect the life expectancy through better nutrition, more healthy life style and access to better health care services. Preston (16) examined the interplay between income and life expectancy and developed the Preston curve for the interplay between income and life expectancy. The Preston curve indicated that the individuals in higher-income countries usually have higher life span than those from lower-income countries. However, the increases in income lead to higher development in life expectancy at low values of GDP *per capita*, but the increases in life expectancy reduce at greater values of GDP *per capita*. In addition, the studies document that people in low-income countries usually get more of communicable and noncommunicable diseases and injuries as a consequence of poor nutrition, anxiety, stress, unemployment, and unhealthy life style (17, 18). Therefore, income is expected to positively impact life expectancy considering the aforementioned considerations, but income inequality can negatively influence life expectancy by preventing the people’s access to adequate food and healthcare services.

ICT indicators can impact life expectancy by means of direct and indirect aspects. First, ICT indicators can improve life expectancy through easing the access to health-related information and sharing of information about health, healthy nutrition, and epidemics (19–21). Secondly, ICT indicators can also improve health awareness of individuals and doctor-patient communication, and, in turn, increase the early detection and treatment of diseases and lead individuals to make informed decisions about life quality (22, 23). Thirdly, ICT indicators can negatively impact life expectancy through insufficient physical activities (24, 25). Last, ICT indicators can influence life expectancy indirectly via economic growth, unemployment, and development of green technologies (26–28). Consequently, ICT indicators are expected to have a significant influence on life expectancy based on country own characteristics. Last, CO_2_ emissions have serious negative effects on the population health through leading to dizziness, confusion, unconsciousness and death and in turn CO_2_ emissions are expected to negatively impact life expectancy (29, 30).

This article explores the effect of education and income inequalities, ICT indicators, CO_2_ emissions, real GDP *per capita* on life expectancy and infant mortality rate in the recent EU members. The recent EU countries have implemented a remarkable social, economic and institutional achievements as of early 1990s as a result of transition and EU membership processes and in turn remarkable progress in life expectancy. Therefore, we determined our sample as the EU transition countries. The associated empirical studies have mainly questioned the effect of income and education levels on life expectancy. Also, the studies have usually examined lifespan gap among the persons with different income and education levels. For these reasons, we examine the effect of inequalities in education and income on life expectancy to make an empirical contribution to the present literature. Furthermore, the effect of ICT indicators and CO_2_ emissions on life expectancy has been little explored until now. In this regard, the second empirical contribution of the study is to investigate the effect of ICT indicators and CO_2_ emissions on life expectancy.

The next part of the article presents an extensive empirical literature summary about the interplay among educational inequality, income inequality, ICT, and life expectancy; then, the dataset and regression analysis are described; estimation of regression analysis and discussion are introduced, and the article eventuates in the Conclusion.

## 2 Literature review

Life expectancy is among the most widespread health status indicators and improvements in life expectancy also indirectly indicate the improvements in living standards and economic development of a country. On the other hand, a healthy society is one of the key prerequisites for economic growth and development. Therefore, drivers of life expectancy have been addressed by the academicians on a large scale and great numbers of demographic, institutional, socio-economic, environmental, cultural, and political factors have been suggested to improve the life expectancy or life expectancy differences between countries (7–9, 31–34). This study investigates the effect of inequalities in education and income, ICT indicators, CO_2_ emissions, and real GDP *per capita* on life expectancy seeing the associated empirical literature on determinants of life expectancy.

In the empirical literature, the academicians have usually examined the interplay between education indicators and life expectancy and a positive influence of various education indicators on life expectancy has been uncovered mostly (34–37). However, few scholars have studied the effect of educational inequalities on lifespan and revealed the educational inequality as significant factor of differences in life expectancy in different countries (38–49). These studies have extensively examined the gap in life expectancy among individuals with diverse education levels (38–49).

Kalediene and Petrauskiene (36) investigated the educational inequalities in life expectancy in Lithuania based on data from 1989 census and found that the men and women with primary or lower education, respectively, had a shorter lifespan of 11.7 and 4.3 years than that of the persons with university education and educational inequalities had relatively more impact on the mortality of the population between the ages of 25–44. Renard et al. (39) explored the course of educational inequalities in disability-free life expectancy and life expectancy in Belgium over the 2001–2011 term and uncovered that life expectancy was increased in all education levels, but the increases in life expectancy were higher in the highest education level in both men and women.

Mackenbach et al. (40) studied the drivers of inequalities in life expectancy in 15 European countries and discovered a notable gap in life expectancy of high- and low-educated persons. Solé-Auró et al. (41) examined the educational inequalities in life expectancy in Spain in 2012 and revealed the educational inequality as significant factor of differences in life expectancy. Welsh et al. (42) examined the inequalities in life expectancy in Australia by education levels through data of 2016 Australian Census and discovered the significant inequalities in life expectancy due to education.

Mesceriakova-Veliuliene et al. (42) studied the effect of education over inequalities in life expectancy at 30 years old in Lithuania over the 2001–2014 through regression and revealed that life expectancy at 30 years old in men and women with post-secondary education was greater than in the individuals with up-to-secondary education. Danler and Pfaff (44) questioned the influence of educational inequality on inequalities in life expectancy in 31 European countries for the period of 1970–2010 via regression and their obtained findings revealed a positive interplay between inequalities in life expectancy and educational inequality.

Murtin et al. (45) examined the inequalities in life expectancy in 23 OECD countries in 2011 and disclosed a gap of 7.6 (men) and 4.8 (women) years in life expectancy between high and low-educated people at age 25 years, and a gap of 3.6 (men) and 2.6 (women) years in life expectancy between high and low-educated people at age 65. Murtin and Lübker (46) uncovered similar findings for 25 OECD members over the 2013–2019 period. Bartoll-Roca et al. (47) examined the gap in life expectancy by educational level in Barcelona between 2004 and 2018 and revealed a life expectancy gap of 1.93 years for women and 3.08 years for men.

Zazueta-Borboa et al. (48) analyzed the relationship between mortality and education levels in individuals over 30 years of age in England (1972–2017), Finland (1971–2017), Italy (Turin; 1972–2019), and Wales (1972–2017) by regression and their results disclosed an elastic interaction between education inequalities and mortality. Gutacker et al. (49) examined the interaction between quality-adjusted life expectancy and educational attainment in Norway and uncovered that persons with higher educational attainment had relatively longer lives.

Income level is another notable driver of life expectancy and improvements in life expectancy are experienced when living standards are increased as a result of growing income levels (50). Miladinov (51) found a positive relationship between GDP *per capita* and life expectancy in Macedonia, Serbia, Bosnia and Herzegovina, Montenegro, and Albania for the period of 1990–2017. Shkolnikov et al. (52) and He and Li (53) reached the similar results with Miladinov (51).

On the other hand, few researchers have investigated the interplay between life expectancy and income inequality and disclosed a negative effect of income inequality on expected lifespan. In this context, Chetty et al. (54) respectively revealed a 14.6- and 10.1-years gap in life expectancy between the richest 1% and poorest 1% of individuals for men and women by analyzing the yearly data of 1,408,287,218 persons from the US between 1999 and 2014. Ahmad et al. (55) studied the effect of income inequality and urbanization on life expectancy in 6 South Asian states over the 1997–2021 by regression and disclosed a negative effect of income inequality on life expectancy.

ICT indicators have ability to foster life expectancy through simultaneous multiple channels at theoretical terms, but the researchers have begun to question the interplay between ICT and life expectancy during the past few years through regression and typically determined a positive effect of various ICT indicators on life expectancy as in Table 1. In this context, Majeed and Khan (19), Alzaid et al. (56), Shao et al. (26), Ronaghi (57), Mlambo et al. (58), and Byaro et al. (59) found a positive effect of various ICT indicators such as internet usage, mobile subscription, and fixed telephone subscriptions on line expectancy. However, Wang et al. (55) disclosed a positive effect of internet and mobile cellular subscriptions on life expectancy, but a negative effect of fixed telephone subscriptions on life expectancy. Last, Vaidean and Achim (56) unveiled an inverted U-shaped linkage between ICT and life expectancy.

**Table 1:** ICT and life expectancy.

Article	States; study duration	ICT indicators	Effect of ICT indicators on life expectancy
Majeed and Khan (19)	184 states; 1990–2014	Internet users, mobile cellular subscriptions, and fixed telephone subscriptions.	Positive
Alzaid et al. (56)	156 states; 1999, 2005, and 2010	Internet	Positive
Shao et al. (26)	141 states; 2012–2016	ICT environment index, ICT usage index	Positive
Ronaghi (57)	Middle Eastern states; 2008–2018	ICT index	Positive
Mlambo et al. (58)	SADC states; 2000–2018	Mobile cellular telephone subscriptions	A weak positive effect
Byaro et al. (59)	48 sub-Saharan Africa states; 2000–2020	Internet usage	Positive
Wang et al. (60)	28 states, 2000–2017	Mobile internet use, mobile cellular subscriptions, fixed telephone subscriptions	Positive (mobile internet use and mobile cellular subscriptions) and negative (fixed telephone subscriptions)
Vaidean and Achim (24)	185 states; 2005–2018	Internet usage and mobile cellular subscriptions	An inverted U-shaped interplay

Last, the impact of CO_2_ emissions on life expectancy have been investigated by the researchers especially as of 2020 and Das and Debanth (61), Azam and Adeleye (62), and Saidmamatov et al. (63) disclosed a negative impact of CO_2_ emissions on life expectancy, but Mahalik et al. (64) uncovered that the impact of CO_2_ emissions on life expectancy was negative in emerging countries and positive in developing countries.

This study explores the role of inequalities in education and income, ICT indicators, CO_2_ emissions and real GDP *per capita* in the heterogeneity in life expectancy across countries. In this context, following five hypotheses will be questioned in the study based on the associated theoretical views and empirical literature:

*H*1: There is a significant association between educational inequality and life expectancy.

*H*2: There is a significant association between income inequality and life expectancy.

*H*3: There is a significant association between ICT indicators and life expectancy.

*H*4: There is a significant association between CO_2_ emissions and life expectancy.

*H*5: There is a significant association between real GDP *per capita* and life expectancy.

Our expectation about the nexus among life expectancy, infant mortality, inequalities in education and income, ICT indicators, CO_2_ emissions, and real GDP *per capita* based on the related theoretical views in the introduction, empirical findings presented in the literature review and socio-economic characteristics of the recent EU members is as following (Table 2).

**Table 2:** Interaction among life expectancy, infant mortality, inequalities in education and income, ICT indicators, CO_2_ emissions, and real GDP *per capita*.

Independent variables	Life expectancy at birth	Infant mortality rate
Educational inequality	Negative	Positive
Income inequality	Negative	Positive
ICT indicators	Positive	Negative
CO_2_ emissions	Negative	Positive
Real GDP *per capita*	Positive	Negative

## 3 Data and method

This research questions the effect of education and income inequalities, ICT indicators, CO_2_ emissions and real GDP *per capita* on life expectancy in the recent EU members through panel regression analysis. Furthermore, a second model is constructed with infant mortality instead of life expectancy to check the robustness of the findings. The variables used in the econometric analysis are reported in Table 3. In this context, the dependent variables of the study are life expectancy and infant mortality rate. Life expectancy and infant mortality are, respectively, represented by life expectancy at birth (years) and infant mortality rate per 1,000 live births. Life expectancy at birth shows how long a newborn infant lives by assuming that prevailing mortality patterns at infant’s birth were to stay the same during its life (5). Therefore, variation in the current mortality patterns can significantly change the life expectancy at birth.

**Table 3:** Data description.

Variable abbreviation	Data definition	Data source
LIFEXP	Life expectancy at birth (years)	UNDP (5)
INFMORT	Infant mortality rate (per 1,000 live births)	World Bank (65)
EDINEQ	Educational inequality index	UNDP (5)
INCOMINEQ	Income inequality index	UNDP (5)
INET	Individuals using the Internet (% of population)	World Bank (66)
MOBILE	Mobile cellular subscriptions (per 100 people)	World Bank (67)
CO2	Total CO emissions *per capita* (tCO_2_ equivalent *per capita*)	European Environment Agency (68)
RGDP	Real GDP *per capita* (constant 2015 US$)	World Bank (69)

On the other hand, inequalities in education and income are, respectively, proxied by indices of education and income inequalities and ICT is represented by internet usage rate and mobile cellular subscriptions. Last, CO emissions *per capita* and real GDP *per capita* are included in the regression analysis as the control variables. The variables of life expectancy and inequalities in education and income are obtained from the database of UNDP (5). UNDP (5) makes use of Atkinson (70) to calculate the inequalities in education and income. The inequality measure of A is 1−g/μ where μ and g are, respectively, the arithmetic and geometric means of the distribution. In this case, A can be expressed as following (Equation 1):


(1) $$A_{x}=1-\frac{\sqrt[{n}]{X_{1}\ldots .X_{n}}}{\bar{X}}\;$$

On the other hand, infant mortality rate, ICT indicators of internet usage and mobile subscriptions, and real GDP *per capita* are obtained from the database of World Bank. Last, CO_2_ emissions are provided from the database of European Environment Agency.

The effect of education and income inequalities, ICT indicators, CO_2_ emissions, and real GDP *per capita* on life expectancy and infant mortality rate is analyzed in the sample of 11 recent EU members including Bulgaria, Croatia, Czechia, Estonia, Latvia, Lithuania, Poland, Romania, Slovakia, and Slovenia. UNDP (5) began to calculate inequalities in education and income as of 2010 and all variables under consideration have existed until 2022. Therefore, it led us to conduct the econometric analysis between 2010 and 2022. Only internet usage data for Slovakia in 2022 is missing and all other variables for the 2010–2022 period are complete. In the analyses, the logarithmic forms of the variables are used to eliminate the seasonality. Empirical analyses are conducted using Stata 17.0.

Tables 4, 5 respectively report the panel and country-level summary statistics of LIFEXP, INFMORT, EDINEQ, INCOMINEQ, INET, MOBILE, CO2, and RGDP. The arithmetic means of LIFEXP, INFMORT, EDINEQ, INCOMINEQ, INET, MOBILE, CO2, and RGDP for the overall panel are, respectively, 76.555 years, 4.248 per 1,000 live births, 16.599, 3.715, 74.456% of population, 118.908 subscriptions per 100 people, 6.560 CO emissions *per capita* and USD14680.68 *per capita*. However, the variables of RGDP, MOBILE, and INET exhibit a remarkable variation among the countries as seen in Table 5, but the variables of LIFEXP, INFMORT, EDINEQ, INCOMINEQ, and CO2 show a moderate variation among the countries between 2010 and 2022.

**Table 4:** Panel-level data summary statistics (2010–2022).

Summary statistics	LIFEXP	INFMORT	EDINEQ	INCOMINEQ	INET	MOBILE	CO2	RGDP
Mean	76.555	4.248	16.599	3.715	74.456	118.908	6.560	14680.68
Median	76.457	4.000	16.856	3.194	75.830	118.107	6.114	14774.99
Maximum	82.133	10.50	26.711	7.307	91.179	163.131	14.900	25349.76
Minimum	71.528	1.50	9.059	1.229	39.930	65.987	3.528	6434.552
Std. Dev.	2.106	1.822	4.682	1.799	11.193	16.482	2.712	4283.003
Skewness	0.395	1.061	0.139	0.318	−0.808	−0.359	1.229	0.107
Kurtosis	2.791	1.338	1.889	1.728	3.477	4.175	4.024	2.528

**Table 5:** Country-level data summary statistics (2010–2022).

Countries	Summary statistics	LIFEXP	INFMORT	EDINEQ	INCOMINEQ	INET	MOBILE	CO2	RGDP
Bulgaria	Mean	74.069	6.708	23.176	6.4366	60.911	125.7435	6.3903	7501.740
	Median	74.4820	6.400	23.7156	6.4334	59.826	123.8463	6.3720	7344.443
	Maximum	75.06	9.00	26.34	7.31	79.13	143.97	7.24	9550.94
	Minimum	71.53	5.00	18.52	5.77	46.23	113.85	5.29	6434.55
	Std. Deviation	1.14867	1.360	2.52119	0.47043	10.282	10.71162	0.51259	926.134
	Skewness	−1.632	0.448	−0.502	0.357	0.312	0.567	−0.383	0.898
	Kurtosis	1.695	−1.176	−1.007	−0.551	−0.859	−1.218	0.748	0.324
Croatia	Mean	77.916	4.177	15.835	5.1350	70.558	108.353	4.4319	13084.485
	Median	77.953	4.100	15.841	4.9891	69.845	106.806	4.3840	12617.306
	Maximum	79.24	4.70	17.47	6.75	82.07	117.54	4.89	16610.32
	Minimum	76.81	3.90	13.54	3.94	56.55	102.94	4.17	11703.98
	Std. Deviation	0.647	0.242	1.452	0.91409	8.521	4.575	0.222	1569.388
	Skewness	0.357	0.902	−0.407	0.588	−0.258	0.755	0.941	1.232
	Kurtosis	0.347	0.219	−1.474	−0.584	−1.023	−0.404	0.330	0.777
Czechia	Mean	78.426	2.477	10.8880	1.3185	77.081	124.661	10.012	18300.221
	Median	78.575	2.500	11.1095	1.3192	76.481	124.338	10.070	18247.011
	Maximum	79.24	2.70	11.36	1.39	84.54	132.29	11.31	20237.29
	Minimum	77.57	2.10	9.97	1.23	68.82	118.35	8.63	16505.95
	Std. Deviation	0.553	0.179	0.5631	0.05107	4.825	4.50385	0.773	1504.855
	Skewness	−0.112	−1.042	−0.677	−0.298	−0.157	0.125	−0.137	0.051
	Kurtosis	−1.418	0.227	−1.401	−1.032	−0.963	−1.016	−0.257	−1.830
Estonia	Mean	77.533	2.354	16.632	2.2726	85.202	89.085	12.053	18257.411
	Median	77.656	2.300	15.674	2.3120	88.102	87.088	13.430	17945.945
	Maximum	79.16	3.60	19.77	2.57	91.02	122.17	14.90	21707.24
	Minimum	75.75	1.50	14.61	1.91	74.10	65.99	6.92	14585.84
	Std. Deviation	0.958	0.691	1.982	0.21386	5.922	17.625	2.82432	2221.275
	Skewness	−8.162	0.447	0.810	−0.405	−0.841	0.801	−0.869	0.009
	Kurtosis	−8.377	−0.935	−0.858	−1.011	−0.852	−0.038	−0.961	−1.125
Hungary	Mean	75.526	4.085	13.121	3.2195	77.004	109.135	4.8398	13337.635
	Median	75.681	4.000	12.499	3.1940	76.074	105.969	4.8310	13035.353
	Maximum	76.45	5.10	15.62	4.00	90.46	120.28	5.20	16336.24
	Minimum	74.52	3.30	11.34	2.66	65.00	100.57	4.40	11307.72
	Std. Deviation	0.6417	0.6731	1.588	0.41401	7.629	7.619	0.247	1691.271
	Skewness	−0.349	0.239	0.756	0.746	0.366	0.399	−0.555	0.377
	Kurtosis	−1.132	−1.604	−0.847	0.034	−0.486	−1.826	−0.465	−1.172
Latvia	Mean	74.518	4.30	20.054	2.7161	80.179	118.421	3.778	14142.249
	Median	74.685	4.00	20.127	2.8301	79.842	117.096	3.703	14242.573
	Maximum	75.93	6.50	21.84	3.24	91.18	134.29	4.09	17081.34
	Minimum	72.95	2.80	18.95	1.84	68.42	106.95	3.53	10962.23
	Std. Deviation	0.867	1.182	0.864	0.43020	7.655	9.479	0.178	1911.754
	Skewness	−0.139	0.581	0.392	−0.598	0.045	0.297	0.783	−0.184
	Kurtosis	−0.647	−0.772	−0.215	−0.343	−1.113	−1.443	−0.445	−0.980
Lithuania	Mean	74.662	3.677	19.976	4.8160	75.074	143.858	4.658	15035.082
	Median	74.610	3.800	19.796	4.4880	74.377	142.494	4.620	14810.252
	Maximum	76.21	4.90	22.48	6.98	87.72	163.13	4.95	18535.08
	Minimum	73.42	2.90	16.46	2.91	62.12	130.02	4.39	11106.95
	Std. Deviation	0.802	0.665	1.7828	1.39084	8.473	11.258	0.181	2418.2027
	Skewness	0.381	0.269	−0.350	0.318	0.012	0.317	0.204	−0.041
	Kurtosis	−0.349	−0.990	−0.567	−1.196	−1.173	−1.095	−1.211	−1.202
Poland	Mean	77.1623	4.223	18.028	5.1255	72.830	133.851	8.534	13340.293
	Median	76.996	4.100	18.841	4.9374	73.301	131.944	8.5350	12936.573
	Maximum	77.93	5.10	19.80	6.18	86.94	147.57	8.88	17117.33
	Minimum	76.32	3.80	14.82	4.15	61.95	121.65	7.99	10755.66
	Std. Deviation	0.543	0.449	1.771	0.657	9.377	8.326	0.28393	1975.908
	Skewness	−0.082	0.830	−0.873	0.250	0.180	0.446	−0.490	0.490
	Kurtosis	−1.460	−0.543	−1.000	−1.237	−1.604	−0.828	−0.724	−0.798
Romania	Mean	75.002	7.408	22.761	5.7173	61.582	115.987	4.0533	9690.692
	Median	74.928	7.100	22.765	5.6684	59.50	115.824	3.9730	9286.596
	Maximum	76.51	10.50	26.71	6.79	85.50	119.79	4.58	12188.64
	Minimum	74.00	5.30	20.63	5.17	39.93	113.42	3.83	7657.54
	Std. Deviation	0.811	1.872	1.604	0.444	15.784	1.901	0.23150	1523.206
	Skewness	0.589	0.395	1.051	1.215	0.134	0.655	1.458	0.259
	Kurtosis	−0.713	−1.391	2.055	1.657	−1.279	−0.095	1.476	−1.463
Slovakia	Mean	76.486	5.169	11.364	1.757	81.295	124.42	6.464	16673.555
	Median	76.649	5.100	11.088	1.748	80.449	128.699	6.473	16687.984
	Maximum	77.69	5.70	13.99	2.22	90.23	135.67	7.14	18877.96
	Minimum	74.91	4.90	9.41	1.43	74.44	109.80	5.72	14584.26
	Std. Deviation	0.849	0.275	1.483	0.26042	5.342	9.985	0.41288	1477.207
	Skewness	−0.548	0.749	0.191	0.671	0.723	−0.369	−0.240	0.024
	Kurtosis	−0.728	−0.556	−1.302	−0.340	−0.667	−1.737	0.046	−1.528
Slovenia	Mean	80.798	2.146	10.747	2.3506	77.293	114.4686	6.9478	22124.161
	Median	80.822	2.100	10.457	2.1236	75.499	114.1500	7.0010	21541.176
	Maximum	82.13	2.60	12.18	3.18	89.00	126.18	8.04	25349.76
	Minimum	79.70	1.90	9.06	1.99	67.34	103.14	6.01	19922.47
	Std. Deviation	0.685	0.237	1.014	0.42402	7.682	6.82110	0.66746	1867.789
	Skewness	0.265	0.770	0.205	1.079	0.379	−0.022	0.284	0.495
	Kurtosis	−0.258	−0.623	−0.990	−0.588	−1.29	−0.600	−0.893	−1.247

The effect of education and income inequalities, ICT indicators, CO_2_ emissions, and real GDP *per capita* on life expectancy in the sample of the recent EU members is analyzed within the scope of the model described in Equation 2 through regression analysis. Furthermore, a second model in Equation 3 is established with infant mortality rate as dependent variable to check the robustness of the Model-1. The explained variable is life expectancy (LIFEXP), and explanatory variables are educational inequality (EDINEQ), income inequality (INEQ), internet usage (INET), mobile cellular subscriptions (MOBILE), CO_2_ emissions (CO2), and real GDP *per capita* and ICT development (ICT).


(2) $$\begin{array}{l} LIFEXP_{it}=\alpha_{i}+\beta_{1}EDINEQ_{it}+\beta_{2}INCOMINEQ_{it}\\ \;\;\;\;\;\;\;\;\;\;\;\;\;\;\;\;\;\;\;\;\;\;\;+\beta_{3}INET_{it}+\beta_{4}MOBILE_{it}+\beta_{5}CO2_{it}\\ \;\;\;\;\;\;\;\;\;\;\;\;\;\;\;\;\;\;\;\;\;\;\;+\beta_{6}RGDP_{it}+\varepsilon_{it} \end{array}$$

(3) $$\begin{array}{l} INFMORT_{it}=\alpha_{i}+\beta_{1}EDINEQ_{it}+\beta_{2}INCOMINEQ_{it}\\ \;\;\;\;\;\;\;\;\;\;\;\;\;\;\;\;\;\;\;\;\;\;\;\;\;\;\;\;\;\;+\beta_{3}INET_{it}+\beta_{4}MOBILE_{it}+\beta_{5}CO2_{it}\\ \;\;\;\;\;\;\;\;\;\;\;\;\;\;\;\;\;\;\;\;\;\;\;\;\;\;\;\;\;+\beta_{6}RGDP_{it}+\varepsilon_{it} \end{array}$$

where i and t, respectively, represents the countries and years of 2010–2022.

## 4 Results

In the results section, first the presence of collinearity is examined. In this context, the correlation matrix among the explanatory variables and variance inflation factor (VIF) scores are calculated and shown in Table 6. A VIF value that is higher than 5 or 10 denotes a problem of collinearity (71). Therefore, the low correlation and the VIF values in Table 5 denote the non-existence of the collinearity problem.

**Table 6:** Correlation matrix among variables.

	EDINEQ	INCOMINEQ	INET	MOBILE	CO2	RGDP	VIF
EDINEQ	1						3.11
INCOMINEQ	0.489 (0.000)	1					1.98
INET	−0.381 (0.002)	−0.205 (0.004)	1				2.46
MOBILE	−0.291 (0.0016)	−0.354 (0.011)	0.346 (0.008)	1			2.19
CO2	−0.186 (0.005)	−0.101 (0.006)	−0.261 (0.000)	−0.174 (0.010)	1		3.09
RGDP	−0.413 (0.000)	−0.422 (0.000)	0.308 (0.000)	0.215 (0.000)	0.397 (0.004)	1	2.88

Then, cross-sectional dependence (CD) and heterogeneity are explored through CD and delta tilde tests in Tables 6, 7. The presence of CD among the variables in the models defined in Equations 2, 3 is questioned by LM, LM CD, and LM_adj._ tests and their results are introduced in Table 7. The null hypothesis in terms of CD independence is declined in both models because the probability values of the three tests are lower than 5%. In conclusion, CD among these variables in both models is disclosed.

**Table 7:** Results of CD tests.

Cross-section dependence tests	Model-1		Model-2	
	Test statistics	Probability values	Test statistics	Probability values
LM	56.493	0.000	34.291	0.000
LM CD	59.125	0.014	36.908	0.000
LM_adj._	60.336	0.009	38.045	0.000

The presence of homogeneity is explored by means of two delta tilde tests in both models, and their results are denoted in Table 8. The null hypothesis in terms of homogeneity is declined for both tests and the availability of heterogeneity is disclosed in both models.

**Table 8:** Results of delta tilde tests.

Test	Model-1		Model-2	
	Test statistic	Probability values	Test statistic	Probability values
Δ̃	24.382	0.000	16.326	0.003
Δ̃_adj._	27.404	0.000	18.288	0.001

The stationarity of LIFEXP, INFMORT, EDINEQ, INCOMINEQ, INET, MOBILE, CO2 and RGDP is explored by Pesaran (72) CADF unit root test in deference to the presence of CD among the series and the test statistics are reported in Table 9. The results of the CADF unit root test denote that the series under consideration are not stationary at the level, but these series have become stationary at the first-differenced values.

**Table 9:** Results of CADF unit root test.

Variables	Level		First differences of the variables	
	Constant	Constant + Trend	Constant	Constant + Trend
LIFEXP	−1.107	−1.234	−8.201**	−8.693**
INFMORT	−0.763	−0.861	−7.915**	−8.667**
EDINEQ	−0.829	−0.944	−9.645**	−10.453**
INCOMINEQ	−0.913	−1.109	−8.312**	−9.217**
INET	−1.366	−1.453	−8.504**	−9.505**
MOBILE	−1.204	−1.358	−7.584**	−8.534**
CO2	−0.945	−1.114	−9.003**	−9.991**
RGDP	−0.821	−0.926	−8.547**	9.705**

**significant at 5% level [The null hypothesis (There is unit root) is rejected.].

Panel regression can be estimated through pooled, fixed, and random effects (73). Chow (74), Breush-Pagan (BP) (75), and Hausman (76) tests are conducted to make a selection among three estimation methods for Model-1 and Model-2 and their results are reported in Table 10. The null hypotheses of Chow and BP tests are declined and fixed and random effects models against pooled regression are specified as the efficient estimation models. Therefore, Hausman (76) test is performed to make a choice between fixed and random effects models and the null hypothesis (there are random effects) is declined and fixed effects model is found to be efficient in both Model-1 and Model-2.

**Table 10:** Results of estimation model selection tests.

Tests		Model-1	Model-2
		*p* value	*p* value
Chow (H0: Pooled regression is efficient against fixed effects model.)		0.000	0.000
BP (H0: Pooled regression is efficient against random effects model.)		0.009	0.000
Hausman	Cross-section random	0.001	0.006
	Period random	0.000	0.011
	Cross-section and period random	0.015	0.000

The econometric models in Equations 2, 3 are estimated through fixed effects model and the estimated parameters are reported in Table 11. The coefficients of the variables in Model-1 uncover that inequalities in education and income, and CO_2_ emissions negatively impact life expectancy, but ICT indicators of internet usage and mobile cellular subscriptions and real GDP *per capita* positively affect life expectancy. Furthermore, real GDP *per capita* has the largest impact on life expectancy when compared with that of other explanatory variables in the Model-1. Furthermore, the coefficients of the variables in Model-2 support the findings of Model-1, because the results of Model-2 indicate that inequalities in education and income and CO_2_ emissions have a positive impact on infant mortality rate. In other words, inequalities in education and income and CO_2_ emissions negatively affect population health. However, ICT indicators of internet usage and mobile cellular subscriptions and real GDP *per capita* have a negative effect on infant mortality rate (positive effect on population health). Similarly real GDP *per capita* has the largest impact on population health. Last, problems of autocorrelation and heteroscedasticity in the estimated models are checked by Wooldridge (77) autocorrelation test and Greene (78) heteroscedasticity test and the findings are found to be robust in terms of autocorrelation and heteroskedasticity problems.

**Table 11:** Results of regression estimation.

Explanatory variables	Coefficients		Std. Error		t-statistic		*p* value	
	Model-1	Model-2	Model-1	Model-2	Model-1	Model-2	Model-1	Model-2
DLnEDINEQ	−0.038	0.028	0.007	0.009	−5.429	3.111	0.003	0.014
DLnINCOMINEQ	−0.051	0.046	0.011	0.005	−4.636	9.200	0.000	0.000
DLnINET	0.029	−0.017	0.008	0.002	3.625	−8.500	0.015	0.000
DLnMOBILE	0.021	−0.011	0.005	0.004	4.201	−2.750	0.007	0.009
DLnCO2	−0.018	0.013	0.003	0.002	−5.667	6.500	0.000	0.004
DLnRGDP	0.063	−0.054	0.015	0.017	4.133	−3.176	0.002	0.000
Constant	1.493	0.986	0.184	0.216	8.114	4.565	0.000	0.000
Model-1				Model-2				
R^2^ = 0.612 F_ist_ = 54.27\u00B0F(p) = 0.000 DW = 2.09 **Diagnostic tests:** Wooldridge autocorrelation test (*p* value) = 0.138 Greene heteroscedasticity test (*p* value) = 0.171				R^2^ = 0.593 F_ist_ = 43.21\u00B0F(p) = 0.000 DW = 2.13 **Diagnostic tests:** Wooldridge autocorrelation test (*p* value) = 0.215 Greene heteroscedasticity test (*p* value) = 0.196				

D and LN, respectively, indicates the first-differenced values and logarithmic forms of the variables.

## 5 Discussion

Education and income have been documented as the dominant drivers of life expectancy in the related literature (12, 79) because both education and income can simultaneously impact life expectancy through multiple different channels. In this context, education can make a positive contribution to life expectancy through increasing health awareness, life quality of the persons, and access to better medical care services. Therefore, inequalities in education can negatively impact health awareness and life quality of the individuals and prevent them to receive adequate medical care services and in turn lead inequalities in life expectancy. Hence, the globally strong positive correlation between inequalities in education and inequalities in life expectancy support these theoretical views (5). Furthermore, Kalediene and Petrauskiene (38), Renard et al. (39), Mackenbach et al. (40), Solé-Auró et al. (41), Welsh et al. (42), Mesceriakova-Veliuliene et al. (43), Danler and Pfaff (44), Murtin et al. (45) discovered a lifespan gap between high- and low-educated individuals. In conclusion, the associated theoretical views and empirical results indicate us that inequalities in education foster the inequalities in life expectancy. Our results also are evaluated to be compatible with these theoretical considerations and results of the empirical studies of (38–45).

On the other hand, income is another dominant factor impacting the life expectancy, because income level can enhance the life expectancy through increasing the access to sources for a healthier life style, better health care services and higher quality educational opportunities. The findings indicate that persons from low-income states often catch communicable and non-communicable diseases and injuries as a consequence of poor nutrition, anxiety, stress, unemployment, and unhealthy life style (17, 18). As a consequence, people from countries with relatively lower income generally have lower expectancy. Furthermore, inequalities in life expectancy in these countries have been relatively higher (5). Chetty et al. (54) and Ahmad et al. (55) also uncovered a significant negative influence of income inequality on life expectancy for the US and South Asian countries, respectively. Furthermore, our results show that real GDP *per capita* and income inequality, respectively, have the largest impact on life expectancy and infant mortality when compared that of ICT indicators and CO_2_ emissions. Consequently, our results line up with the theoretical and empirical literature.

ICT is another key factor which can impact life expectancy through increasing health awareness, healthy nutrition, and inadequate physical activity, digital addiction, and cyber security problems and fostering economic growth and development, unemployment, education, environment, and green technological development. Consequently, the net effect of ICT indicators on life expectancy can theoretically vary of which factors are dominant. However, most of the empirical studies including Majeed and Khan (19), Alzaid et al. (56), Shao et al. (26), Ronaghi (57), Mlambo et al. (58), and Byaro et al. (59) discovered a positive effect of ICT indicators on life expectancy, only Wang et al. (60) has reached different results based on ICT indicators. Our results indicate that ICT indicators of internet usage and mobile cellular subscriptions have a positive impact on life expectancy in compatible with the associated empirical studies.

Last, CO_2_ emissions can negatively impact life expectancy through causing health problems of dizziness, confusion, unconsciousness and death. In the limited empirical literature, Das and Debanth (61), Azam and Adeleye (62), and Saidmamatov et al. (63) uncovered a negative impact of CO_2_ emissions on life expectancy in compatible with theoretical views. However, Mahalik et al. (64) uncovered that the impact of CO_2_ emissions varies among the countries based on countries’ development levels. Our results also indicate that CO_2_ negatively impact life expectancy in parallel with the associated theoretical and empirical literature.

## 6 Conclusion, limitations, and policy recommendations

A healthy society is one of the prerequisites to promote economic growth and development through gains from productivity and education. Therefore, goal of good health and well-being is one of the 17 SDGs and good health is critical for achievement of all other SDGs. In this context, life expectancy is extensively used as an indicator of population health and many demographic, social, economic, cultural, environmental, and political factors have been suggested as the causes of changes in life expectancy. This research investigates the effect of inequalities in education and income, ICT indicators, CO_2_ emissions, and real GDP *per capita* on life expectancy and infant mortality rate in the new EU members which over the 2010–2022 period through panel regression.

The limitations of the study are as following:

Focusing on recent EU members makes the findings ungeneralizable to other regions, or older EU member states with different socioeconomic contexts.

The study is limited to data from 2010 to 2022, because data of inequalities in education and income has existed as of 2010 and all variables under consideration last in 2022. Therefore, changes or trends emerging after 2022 are not captured, which might affect the current relevance of the findings.

The study concentrates on the effect of inequalities in education and income together with ICT indicators and CO_2_ emissions on life expectancy, but does not include demographic factors, healthcare quality, and lifestyle changes.

The presence of cross-sectional dependence can complicate the interpretation of results, as it indicates that unobserved factors affecting one country might influence others.

The results of panel regression for both models reveal that inequalities in education and income, ICT indicators, CO_2_ emissions, and real GDP *per capita* are significant factors for population health. On the one hand, inequalities in education and income and CO_2_ emissions negatively impact life expectancy, but ICT indicators and real GDP *per capita* positively affect the life expectancy. Of all these factors, real GDP *per capita* and income inequality have the highest impact on life expectancy. Last, the results of the second model with infant mortality rate support the results of the first model with life expectancy.

Based on the empirical findings of this paper, four significant policy suggestions are made for progress in life expectancy. First, education policies should be designed to establish equity through distribution of qualified teachers, school funding, and other educational resources and education awareness should be increased in the society through TVs, social media platforms, forums, and community partnerships. Second, income inequality should be reduced via transfers and tax policies. Thirdly, ICT infrastructure and digital literacy are promoted and then many public and private services including healthy nutrition, preventative health care, e-government, e-learning, e-health, and banking should also be given in digital platforms. Fourthly, legal and market based environmental instruments should be taken into consideration for improvement in population health. Future studies can separately study the effect of inequalities in education and income on life expectancy considering the demographic factors and healthcare quality, and lifestyle changes.

## Data Availability

The original contributions presented in the study are included in the article/supplementary materials, further inquiries can be directed to the corresponding author.
